# Wind Turbine Clutter Mitigation in Coastal UHF Radar

**DOI:** 10.1155/2014/529230

**Published:** 2014-01-16

**Authors:** Jing Yang, Chao Pan, Caijun Wang, Dapeng Jiang, Biyang Wen

**Affiliations:** Radar and Signal Processing Lab, School of Electronic Information, Wuhan University, Wuhan 430072, China

## Abstract

Coastal UHF radar provides a unique capability to measure the sea surface dynamic parameters and detect small moving targets, by exploiting the low energy loss of electromagnetic waves propagating along the salty and good conducting ocean surface. It could compensate the blind zone of HF surface wave radar at close range and reach further distance than microwave radars. However, its performance is susceptible to wind turbines which are usually installed on the shore. The size of a wind turbine is much larger than the wavelength of radio waves at UHF band, which results in large radar cross section. Furthermore, the rotation of blades adds time-varying Doppler frequency to the clutter and makes the suppression difficult. This paper proposes a mitigation method which is based on the specific periodicity of wind turbine clutter and performed mainly in the time-frequency domain. Field experimental data of a newly developed UHF radar are used to verify this method, and the results prove its effectiveness.

## 1. Introduction

Coastal area is closely related to the production and living of people. Improvement of capacity in sea state measurement and target detection would enhance the efficiency and safety of marine fishery and engineering, maritime search and rescue, disaster forecasting, and ecological conservation. Compared to conventional measurement devices which directly contact the tested water such as buoys, drifters, and ADCPs (acoustic Doppler current profiler), remote sensing devices are more suitable for the complex and volatile sea environment due to their larger coverage, higher safety, and lower cost in operation. Similar to the HF (high-frequency) surface wave radar which has been widely installed along coastline in many countries, coastal UHF (ultrahigh frequency) radar is based on the electromagnetic wave propagation along the salty and good conducting ocean surface and could monitor areas much larger than the conventional microwave radar coverage. Besides, it could compensate the blind zone of HF radar at close range and provide higher precision data. Several UHF radar hardware systems have been developed and the research of underlying theory is always in progress [1–7].

However, a problem that should not be ignored in the development of coastal UHF radar is the interference from wind turbines that is often called WTC (wind turbine clutter). The interest in renewable energy has grown continuously in recent years due to the climate change and fossil fuel diminishment. As the most mature technology currently available, more and more wind turbines are installed, especially in coastal areas where the wind power is usually rich. These wind turbines cause serious interference to neighbouring radio systems. On the one hand, in order to reach higher altitudes where the winds are more constant and faster, the wind turbine tower and blades are usually tens of meters long, which results in large scattering cross section for radio signals with comparable or shorter wavelength. On the other hand, the rotation of blades adds Doppler frequency to the scattering signals and makes the contamination hard to eliminate. These factors render the installation of wind turbines receiving a lot of objections from managers and operators of radio systems.

The conflict between wind turbines and radio systems has aroused wide attention and several approaches have been proposed to address the problem. From the wind turbine aspect, stealth turbine technology has been investigated, including shaping the wind turbines to avoid specular reflections [8] and partially treating the rotor blades with radar absorbing materials [9]. These methods could indeed reduce the backscattered RCS (radar cross section), but they are generally effective only in specific directions or frequencies, and the cost is high.

From the radar aspect, several signal processing-based mitigation methods have also been proposed. For the air surveillance radar, techniques in development include inhibiting track initiation in the vicinity of wind farms [10], using independent concurrent low and high beam channels to mitigate WTC effect above the wind farm region [11] and installing supplemental radars to complement the wind turbine contaminated area [12]. For the weather radar, mitigation techniques include filtering methods [13, 14], interpolation of spectral moment data over the wind farm using surrounding noncontaminated data [15–18]. Besides, a time-frequency domain method [19] employs the Radon transform to isolate flashes in the spectrogram, and a space domain method [20] utilizes a fully adaptive array to reject the WTC contamination near the ground while preserving scattered energy of the weather above the ground. In addition, a matching pursuit algorithm [21] iteratively removes clutter from multiple wind turbines using the physics-based model of a single wind turbine-derived clutter. These methods are effective, but they are mainly designed for specific radars and not quite appropriate for the coastal UHF radar. For the coastal UHF radar which utilizes backscattered signals from sea surface and vessel targets, WTC may be in the same range-direction cell as the wanted signals; under that condition the space domain mitigation method is not feasible. The interpolation method can be used, but it reduces the radar resolution. The matching pursuit algorithm is not specific to any radar, but its effectiveness has not been proved through actual radar data.

Periodicity is a unique feature of WTC which yet has never been exploited in mitigation algorithms. Method proposed in this paper is based on the periodicity of WTC and performed mainly in time-frequency domain. Field experimental data is used for algorithm verification, which shows that SCR (signal clutter ratio) is substantially increased and the masked ocean wave and vessel echoes could be identified clearly after the mitigation algorithm is performed.

Section 2 presents a short description of the coastal UHF radar.  Section 3 summarizes the characteristics of WTC.  Section 4 details the mitigation algorithm process.  Section 5 demonstrates the results of three validation experiments.  Section 6 concludes the paper.

## 2. UHF Radar Description

The newly developed coastal UHF radar is a coherent Doppler radar with one transmitting channel and eight receiving channels. Different from conventional superheterodyne receivers, the coastal UHF radar receiver directly samples the echoes in RF (radio frequency) stage and both the frequency downconversion and signal demodulation are performed in digital domain and thus the receiver is called “full-digital receiver” [7].

The coastal UHF radar transmits LFMICW (linear frequency modulated interrupted continuous wave) signal which is formed by modulating a LFMCW signal with gating pulses. The transmitting gating pulses are used to isolate the transmitting and receiving phases, which in turn prevent the transmitting signal from jamming the receiver. In the receiver, the dechirp and downconversion processing of receiving signals are performed through CORDIC (coordinate rotation digital computer) algorithm in FPGA (field programmable gates array), and then the resulting data is decimated by CIC (cascade integrator comb) filter to reduce the data rate. After that, the data is sent to PC (personal computer), and the information of range and velocity is extracted through double-FFT (fast Fourier transform) algorithm [22]. The first FFT is performed over each frequency-sweeping period, after which signals of different range bins are separated. The second FFT is done over several continuous-sweeping periods, which realizes frequency bin division and velocity information extraction.

**Figure 1:**
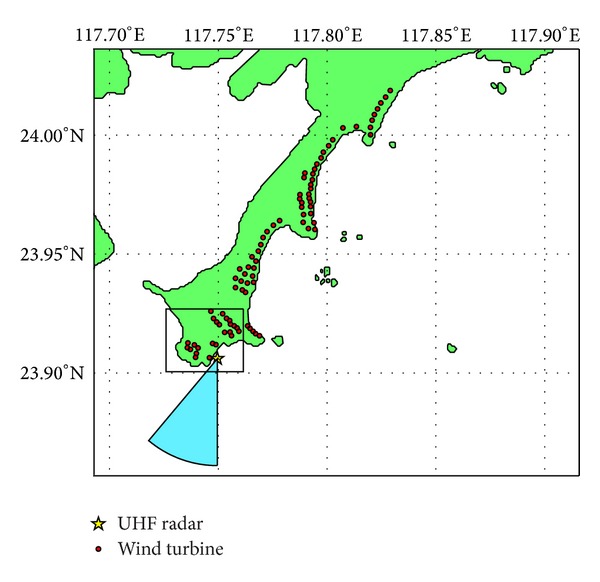
Location of wind turbines and UHF radar.

During the field experimental period, the radar was deployed in LiuAo Peninsula of Fujian Province of China. The main purpose of the experiment was to test the performance of sea state measurement and ship detection. A directional eight-element Yagi antenna was used as the transmitting antenna, and eight identical Yagi antennas formed a 8.4 m-long ULA (uniform linear array) for receiving. The main lobe width of each antenna is 40° in vertical plane and 44° in horizontal plane.  Figure 1 marks the location and main beam coverage of the UHF radar.  Table 1 presents the radar parameters.

## 3. WTC Signature

**Figure 2:**
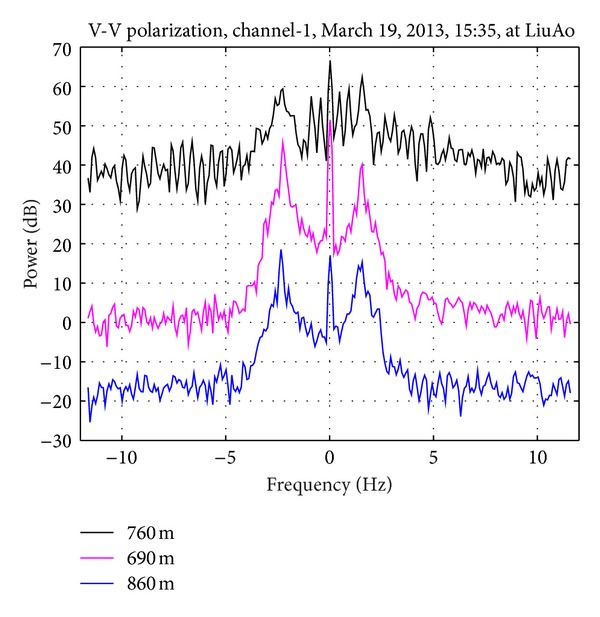
Echo power versus Doppler frequency.

As Figure 1 shows, up to 85 wind turbines are installed in LiuAo peninsula, but none is in the main beam of the UHF radar. However, WTC could still enter the radar through antenna sidelobes. The strong and Doppler frequency domain spreading WTC may mask the echoes from vessels and ocean waves.  Figure 2 presents the PSD (power spectrum density) of three range bins during the same period of time. The curves have been moved vertically for differentiation. The uppermost curve corresponds to a WTC-contaminated range bin, while the below two do not contain WTC. Obviously WTC reduces SNR (signal noise ratio) of Bragg-resonant signals significantly. The aim of the suppression algorithm is to isolate the echoes from vessels and ocean waves from the strong WTC background.

All the turbines in the wind farm are 3-blade horizontal-axis wind turbines with 34 m maximum rotor length and 185.64 Hz maximum Doppler frequency. On the one hand, the blades are longer than the radar range resolution. On the other hand, the maximum Doppler frequency is bigger than the maximum unambiguous Doppler frequency of the UHF radar. Due to the delay-Doppler coupling effect of LFM signal, too high Doppler frequency makes the range measurement error bigger than the range resolution. Thus the WTC may appear in its neighbouring range bins and results in the range aliasing phenomenon.

**Figure 3:**
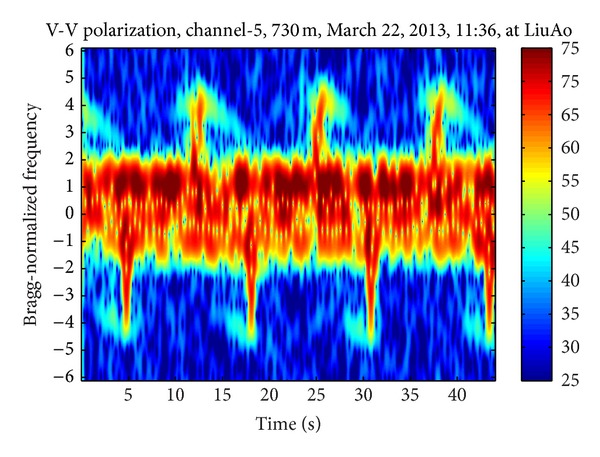
Echo time-frequency spectrogram with no WTC aliasing.

**Figure 4:**
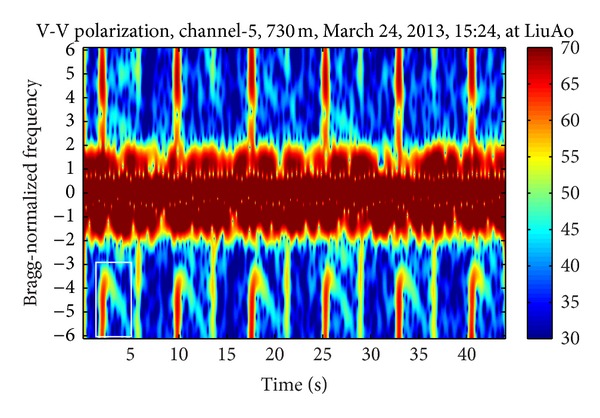
Echo time-frequency spectrogram with WTC aliasing.

**Figure 5:**
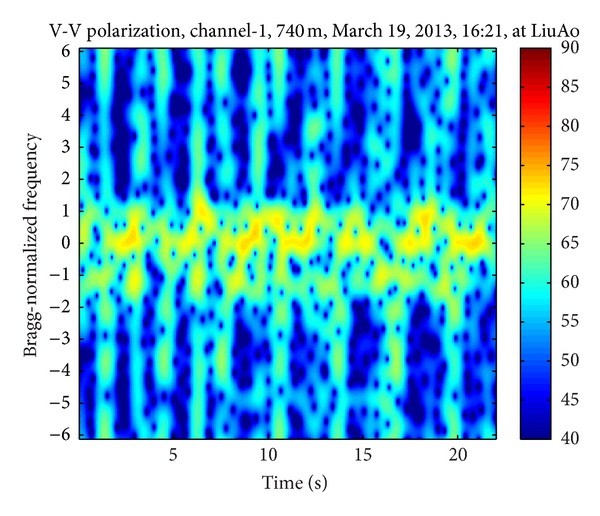
Echo time-frequency spectrogram with WTC aliasing.

Figures 3, 4, and 5 demonstrate three time-frequency spectrograms calculated using STFT (short-time Fourier transform). The frequency axis has been normalized to Bragg frequency and thus the ocean wave echoes concentrate around ±1 in the frequency domain. Obviously the main contribution of WTC is the flashes approximately parallel to the frequency axis, which occur when the blades are orthogonal to the radar line of sight. In Figure 3, the flashes are shorter than the frequency scope, and there is no range aliasing phenomenon. The less powerful curves near the top of the flashes are part of the sinusoid traces of the blade tips, which are too weak to demonstrate their complete shapes. In Figure 4, the flashes exceed the frequency scope and the WTC range aliasing phenomenon occurs. The sinusoid curve in the white rectangular is also caused by the blade tips, but in opposite orientation to those in Figure 3, which is the result of range aliasing effect. The most typical spectrogram is showed in Figure 5, where the range aliasing effect exists and the irregular WTC flashes make it difficult to identify the blade tip sinusoid tracks.

The time-varying radar signature of WTC depends on many factors including wind turbine blade position, yaw motion, and rotation speed. The unstable position and motion parameters as well as the complex aerodynamic shape of blades make it difficult to model WTC with a simple formulation and effectively suppress it. However, the periodicity signature of WTC nearly always exists within a relatively short time, as shown from Figure 3 to Figure 5, even when clutter from several different wind turbines simultaneously enters a single range bin, while the echoes from ocean waves and vessels are not periodic. Thus the periodicity could be used as a significant WTC detection criterion, which is the essence of the method in this paper.

## 4. WTC Mitigation Algorithm

**Figure 6:**
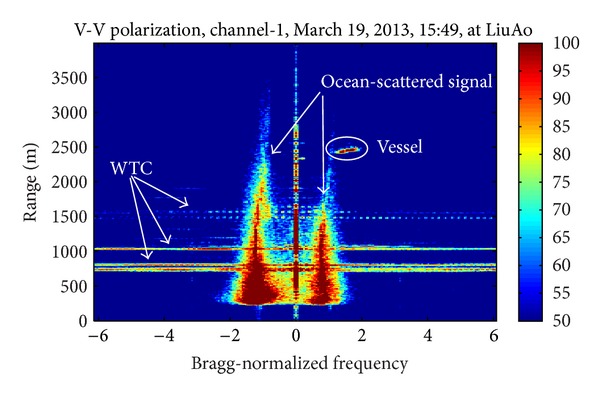
Range-Doppler spectrum of UHF radar.

The mitigation algorithm is performed on each single range bin that has been detected contaminated. The detection method which needs a specific study is not the focus of this paper.  Figure 6 presents a typical Range-Doppler spectrum, from which it is obviously seen that the WTC raises the power of the relatively higher frequency part. A simple method adopted here is to calculate the mean power of each range bin in this frequency part like −6~−3 and 3~6 in Figure 6, and the range bins corresponding to high power values are identified as WTC contaminated. The detailed description of the mitigation algorithm is as follows.

### 4.1. Estimation of WTC Dominant Period

This step is performed in the time-frequency domain, which enables both the frequency-based isolation of ocean wave echoes and time-based period estimation. The process is listed as follows.

**Figure 7:**
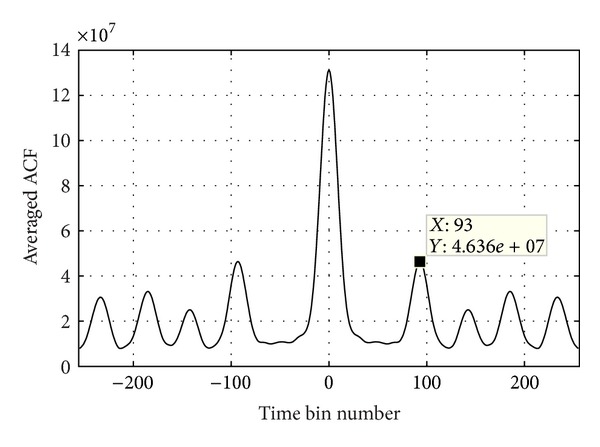
Mean cyclic ACF of all ocean wave echo-free frequency bins in the time-frequency spectrogram.

(1) Calculate the time-frequency spectrogram of the selected range bin using STFT according to
  (1) $$\begin{array}{c} {\text{STFT}}_{s}\left(m,n\right)=\overset{L-1}{\underset{k=0}{\sum }}s\left(k\right)\eta^{*}\left(k-m\right)e^{-j2\pi nFk}, \end{array}$$
  where *s*(*k*) is the signal samples with *L* length. The time interval between each two adjacent samples equates the radar frequency-sweeping period which is denoted by  *T*. *η*(*k*) is the smoothing window with *L*_win_ length. *F* is the interval between frequency bins, which equates 1/*N*_FFT_, where *N*_FFT_ is the number of frequency bins.
(2) Delete the frequency bins where the ocean wave echoes may locate from the spectrogram and calculate the cyclic ACF (autocorrelation function) of amplitude of each remaining frequency bin.
(3) Average all the cyclic ACFs and a curve like Figure 7 is generated.
(4) Find the highest peak with positive time bin number, whose corresponding time bin number *P* is regarded as the WTC period *T*_WTC_ divided by *T*. In Figure 7, *P* = 93, which means that the dominant period of WTC equates 93*T*.

### 4.2. Calculation of the Correlation Function

The correlation function reflects the periodicity strength of each frequency bin at the period *P*. After the WTC period is known, several snapshots of time-frequency spectrogram could be obtained. The spectrogram snapshot means the spectrogram of a section of signal that is shorter than the total signal length *L*. The time interval between snapshots is set as the WTC period *P*, and the time duration of each snapshot is set as a value *L*_snap_ smaller than *L*. Then the number of snapshots *N*_snap_ is obtained according to

(2) $$\begin{array}{c} N_{\text{snap}}=\text{floor}\left(\frac{\left(L-L_{\text{snap}}\right)}{P}\right)+1, \end{array}$$

where floor(*A*) means the maximum integer that is not bigger than *A*.

Process of this step is listed as the following.

**Figure 8:**
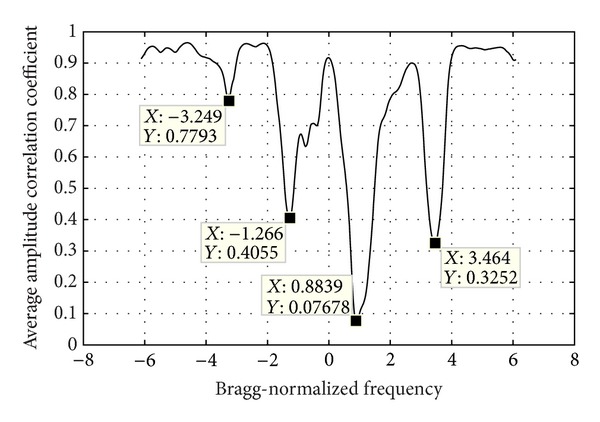
Amplitude correlation versus frequency.

1. Calculate the time-frequency spectrogram of each snapshot using STFT. Both the window length and FFT length should be the same as those of the last step, *L*_win_ and *N*_FFT_.
2. For each frequency bin, calculate the amplitude correlation coefficient between each two spectrogram snapshots. Thus *N*_snap_ snapshots generate *N*_snap_ × (*N*_snap_ − 1)/2 correlation coefficients for each frequency bin.
3. Average all the correlation coefficients corresponding to each frequency bin and then the correlation function like Figure 8 is acquired.

### 4.3. Power Spectrum Density Modulation

WTC possesses strong periodicity, while echoes from vessels and ocean waves do not. The echo of a vessel only exists in a short duration in one range-frequency cell and the ocean wave echo bears stochasticity due to the random motion of ocean waves. Using WTC period as the snapshot interval, the snapshots of spectrogram should be highly correlated with each other if only WTC exists or WTC is predominant in the frequency bin. The vessel and ocean wave echoes could reduce the correlation, and thus the frequency bins where they predominate should have relatively low correlation. Besides, the ambient noise is also random and hence the noise-dominated frequency bins also bear low correlation.

**Figure 9:**
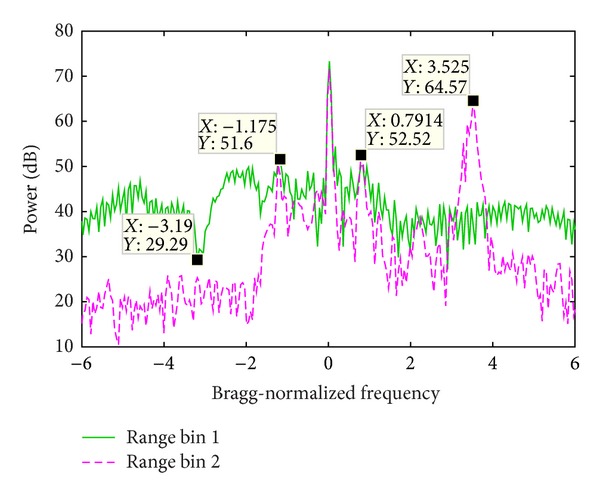
Echo power spectrum density of two range bins.

Figure 8 is the correlation function curve of the data used in Section 5.1, which is composed of signals of two range bins.  Figure 9 shows the PSD of the two range bins. Through comparing Figures 8 and 9, it is inferred that the sink of the correlation function curve at −3.249 Bragg-normalized frequency where the PSD becomes low is caused by noise, the sinks at −1.266 and 0.8839 are the effect of ocean wave echoes, and the vessel echo reduces the correlation at 3.464 Bragg-normalized frequency.

According to the observation result, (3) is proposed to modulate the PSD:

(3) $$\begin{array}{c} S^{\prime }\left(f\right)=S\left(f\right)\times 10^{-aC(f)}, \end{array}$$

where *S*′(*f*) is the PSD after modulation, *S*(*f*) is the original signal PSD, *a* is a positive constant, and *C*(*f*) is the correlation function as shown in Figure 8. Since 10^−*aC*(*f*)^ is a decreasing function, the sinks in the correlation function caused by ocean wave and vessel echoes will increase the PSD amplitude at corresponding positions and hence the wanted signals will be highlighted after modulation. The noise-derived sinks will also raise the PSD, but, since the PSD of the original signal is lower at these positions, it will not stand out after modulation. The constant *a* is chosen mainly based on observations and is set as 5 in this paper.

### 4.4. Multiple Times Processing

Multiple times processing means to repeat the above three steps several times. On the one hand, there may be more than one wind turbine covering a range bin; on the other hand, WTC could spread to multiple range bins due to the delay-Doppler coupling effect of LFM signal as explained in Section 3. Thus one range bin might contain WTC from different wind turbines with different periods. To reach a better result, multiple times processing is necessary.

## 5. Experiments

Data used for the three experiments in this section is from real measured data but with artificial adjustment. The adjustment is to add the signals of two range bins. One is WTC contaminated, while the other is not and the vessel echo is clearly seen in its PSD. The sum signal is then used for algorithm verification. By doing this, the frequency of the vessel echo masked by WTC is known a priori and hence could be used for comparison.

In the three experiments, the signal length *L* equates 512, the smoothing window is Hamming window with length 64, FFT length *N*_FFT_ is set as 512, and the spectrogram snapshot length *L*_snap_ equates 128.

### 5.1. Experiment 1

Figure 9 shows the PSD of two range bin signals, whose sum signal PSD is demonstrated in Figure 11. The range bin 1 is seriously contaminated by WTC and the Bragg peaks are totally buried. The range bin 2 is not contaminated and the Bragg peaks as well as the vessel echo are clearly seen. In order to increase the influence of WTC, during the signal composition, the range bin 1 is weighted by 1 and range bin 2 is weighted by 0.1. In the sum signal, both the Bragg peaks and the vessel echo are masked.

**Figure 10:**
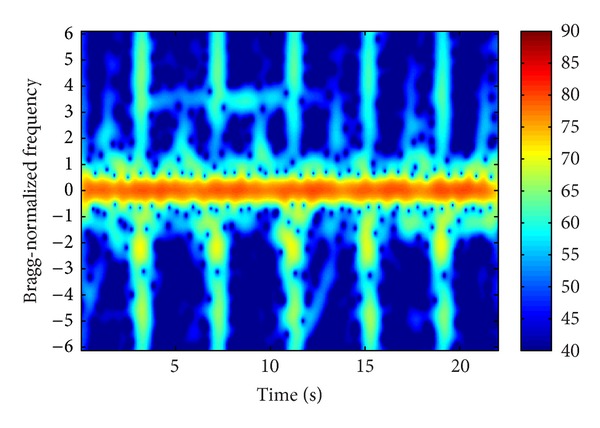
Time-frequency spectrogram of sum signal.

Figure 10 presents the spectrogram of the sum signal. The distribution of WTC blades is regular and the background noise is weak. The vessel echo is between 3 and 13 s at about 3.5 Bragg-normalized frequency.

**Figure 11:**
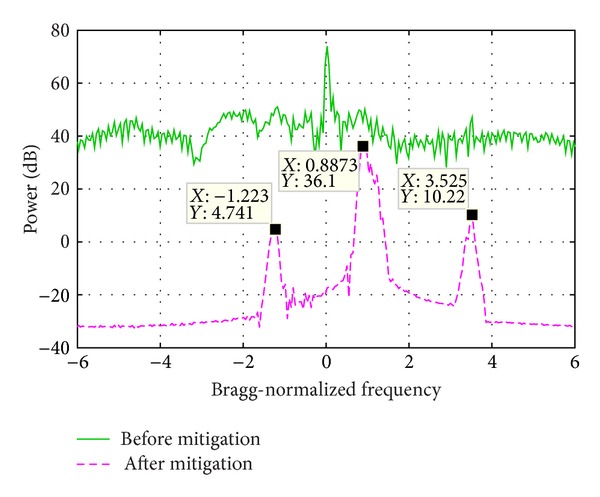
Echo power spectrum density before and after WTC mitigation.

Figure 11 demonstrates the sum signal PSD before and after the mitigation algorithm is performed. Obviously the Bragg peaks and the vessel echoes are prominent in the processing result. The vessel echo frequency is the same as that of range bin 2 as shown in Figure 9. The frequency of the two Bragg peaks is slightly different from that of range bin 2, which might be caused by the distortion of the Bragg peaks of range bin 1.

### 5.2. Experiment 2

**Figure 12:**
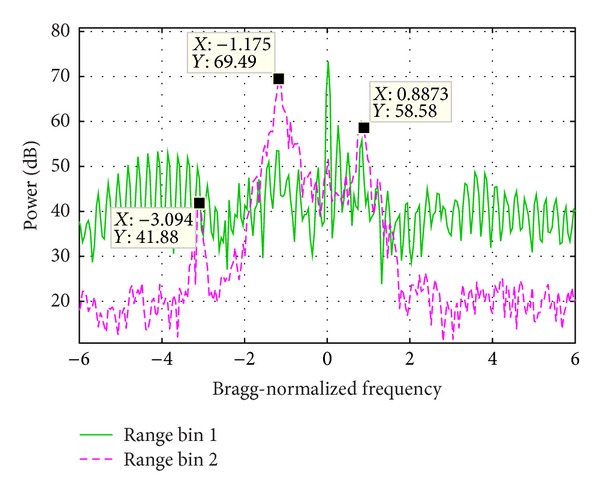
Echo power spectrum density of two range bins.

Figure 12 presents the PSD of two range bins. Range bin 1 is seriously WTC contaminated and range bin 2 is not. The Bragg peaks of range bin 2 are much stronger than the vessel echo. After summation, the Bragg peaks could still be seen, while the vessel echo is masked.

**Figure 13:**
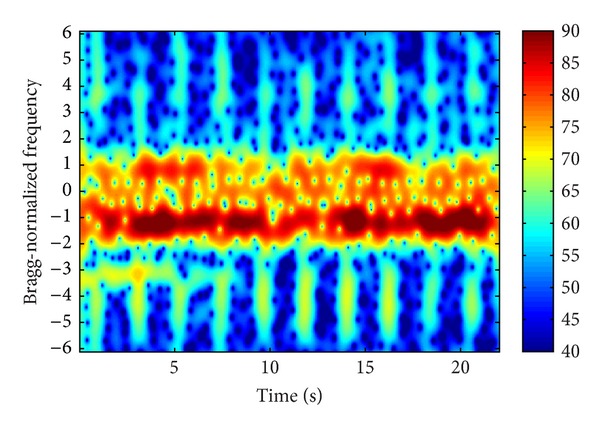
Time-frequency spectrogram of sum signal.

**Figure 14:**
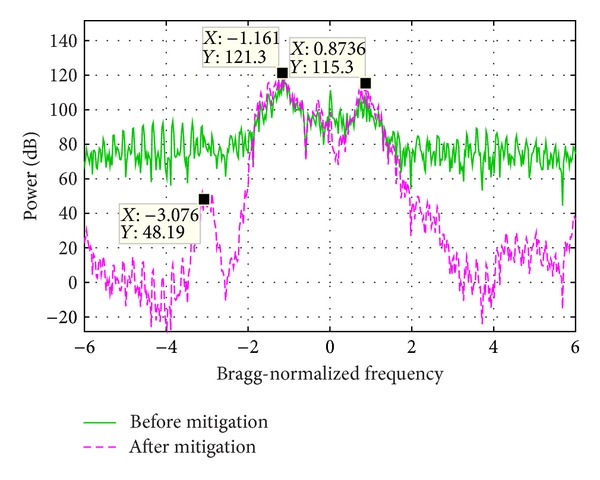
Echo power spectrum density before and after WTC mitigation.

By comparing Figure 13 with Figure 10, it is seen that the background noise is stronger and the WTC is less regular in Experiment 2, and hence the mitigation effect is a little worse than that in Experiment 1. As shown in Figure 14, the vessel echo is clearly seen after algorithm implementation, while only 10 dB than the remaining clutter and noise grounds.

### 5.3. Experiment 3

In the above two experiments, the mitigation algorithm is performed only once. While in this experiment, the algorithm is done twice. Both the Bragg peaks and the vessel echoes are very weak and totally buried in WTC in the original signal. But after the first time mitigation, they become prominent. However, there are also significant peaks at around −2.5 and 2 Bragg-normalized frequency. They are probably caused by the remaining noise since, as shown in Figure 16, frequency bins around these two regions are predominated by noise or very weak WTC. After the second time mitigation, the Bragg peaks and the vessel echo are strengthened further and the unwanted signals at around −2.5 and 2 are weakened.

**Figure 15:**
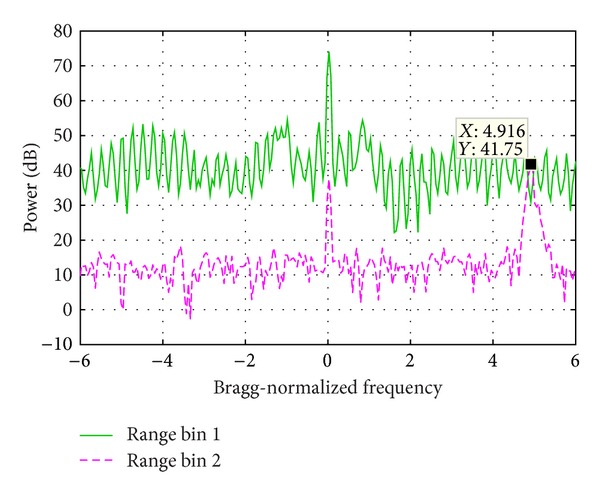
Echo power spectrum density of two range bins.

**Figure 16:**
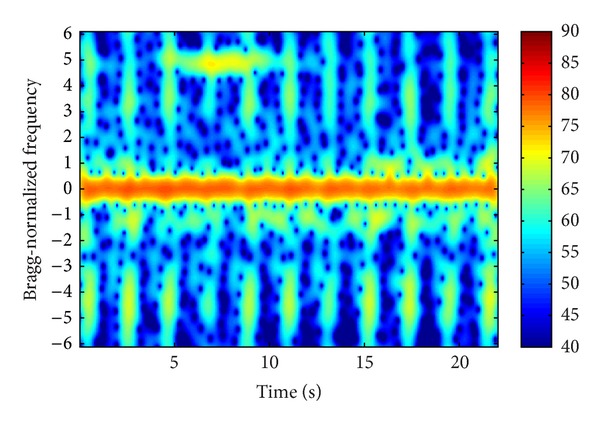
Time-frequency spectrogram of sum signal.

**Figure 17:**
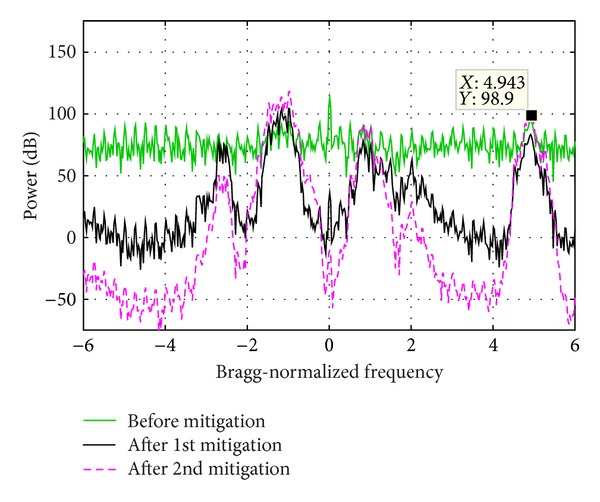
Echo power spectrum density before and after WTC mitigation.

Since the Bragg peaks of the original sum signal are hardly identified, the verification is only performed on the vessel echo, which is proven to be standing out at the right frequency through comparing Figures 15 and 17.

## 6. Conclusion

The complexity of wind turbine motion and blade shape makes the WTC mitigation difficult. The algorithm proposed in this paper utilizes the specific periodicity of WTC which is not possessed neither by the ocean wave and vessel echoes nor by the ambient noise. Three measured data-based experiments are conducted to verify the algorithm performance, which proves that the SNR of the wanted signals, that is, ocean wave and vessel echoes, is significantly increased which could then be easily identified.

The algorithm is not influenced by the range aliasing effect and is capable of addressing the problem that multiple WTC with different periods coexist in one range bin. The optimization of the selection of several parameters in the algorithm, such as the constant in the PSD modulation formulation, still needs further study.

## Figures and Tables

**Table 1:** UHF radar parameters.

Parameter	Value
Carrier frequency	340 MHz
Average transmitting power	25 W
Bandwidth	15 MHz
Frequency-sweeping time width	0.04 s
Frequency-sweeping period	0.043 s
Transmitting pulse period	78.125 *μ*s
Maximum unambiguous range	5.12 km
Range resolution	10 m
Maximum unambiguous Doppler frequency	11.63 Hz
Maximum unambiguous velocity	5.13 m/s
